# How Is Self-Compassion Associated with Prolonged Grief and Posttraumatic Stress After Bereavement? The Mediating Roles of Perceived Stigma and Anger

**DOI:** 10.3390/bs16030354

**Published:** 2026-03-02

**Authors:** Xiaorui Jiang, Zixing Mao, Qinglu Wu, Suqin Tang

**Affiliations:** 1 School of Psychology, Shenzhen University, Shenzhen 518060, China; jiang_xiaorui@126.com (X.J.); maozixing0821@163.com (Z.M.); 2 Institute of Advanced Studies in Humanities and Social Sciences, Beijing Normal University, Zhuhai 519085, China; qinglu-wu@hotmail.com; 3 The Shenzhen Humanities & Social Sciences Key Research Bases of the Center for Mental Health, Shenzhen University, Shenzhen 518060, China

**Keywords:** self-compassion, perceived stigma, self-directed anger, other-directed anger, prolonged grief, posttraumatic stress

## Abstract

**Background:** Self-compassion is negatively associated with stress-related psychopathological symptoms in the grieving process, but the underlying mechanism remains unclear. This study examined the mediating role of perceived stigma and anger in the relationship between self-compassion and symptoms of prolonged grief disorder (PGD) and posttraumatic stress disorder (PTSD) among bereaved individuals. **Methods:** A total of 289 Chinese bereaved adults (70.2% women; Mage = 42.27 years) completed an online survey assessing demographics, loss-related information, self-compassion, perceived stigma, self-directed and other-directed anger, and PGD and PTSD symptoms. Correlation and mediation analyses were conducted. **Results:** Self-compassion was negatively associated with both PGD and PTSD symptoms. Perceived stigma and anger indirectly linked these associations, yet pathways differed regarding anger. Self-compassion was negatively associated with PGD symptoms via self-directed anger, and also indirectly via perceived stigma and self-directed anger. In contrast, self-compassion was negatively associated with PTSD symptoms via other-directed anger, and also indirectly via perceived stigma and other-directed anger. **Conclusions:** Self-directed and other-directed anger play distinct roles linking self-compassion to psychopathological symptoms among bereaved individuals. Cultivating self-compassion may support bereavement adjustment by reducing perceived stigma and anger, and interventions should target specific types of anger based on symptom profiles.

## 1. Introduction

The death of a loved one is one of the most stressful life events (Holmes & Rahe, 1967). While most bereaved people recover from the painful experience with time, a minority of them may continue to suffer from two main clusters of stress-related psychopathological symptoms, including symptoms of prolonged grief disorder (PGD; Eddinger et al., 2021; Komischke-Konnerup et al., 2021; Lundorff et al., 2020) and posttraumatic stress disorder (PTSD; Eddinger et al., 2021; Komischke-Konnerup et al., 2021). PGD, previously known as complicated grief, includes persistent and pervasive grief responses characterized by longing for the deceased or persistent preoccupation with the deceased accompanied by intense emotional pain such as sadness, anger, and blame (World Health Organization, 2018), with a pooled prevalence of approximately 9.8% among adults exposed to bereavement (Lundorff et al., 2020). PTSD is a trauma- and stressor-related disorder that follows exposure to traumatic events, such as bereavement. It is characterized by reexperiencing, avoidance, negative thoughts and feelings, and hyperarousal symptoms (American Psychiatric Association, 2022), with a pooled prevalence of approximately 9.9% in population samples exposed to bereavement (Lechner-Meichsner et al., 2024). A high pooled comorbidity rate of 49% between PGD and PTSD (Komischke-Konnerup et al., 2021) suggests that similar mechanisms may be present in individuals suffering from both disorders (Eddinger et al., 2021). Therefore, it is essential to consider both PGD and PTSD symptoms when investigating psychopathology in bereaved populations. Moreover, the psychological mechanisms underlying PGD and PTSD in bereaved individuals remain underexplored and warrant further investigation.

Research on the mechanism underlying PGD and PTSD has been expanded from merely focusing on risk factors (e.g., pre-loss grief, poor coping skills, and attachment style) to investigating protective factors (Buur et al., 2024). Emotion regulation (ER) may be one of the key protective factors, with evidence showing that difficulties in ER are associated with greater severity of PGD and PTSD (Eisma & Stroebe, 2021; Kennedy et al., 2021; Cesur-Soysal & Durak-Batıgün, 2022). As an adaptive ER strategy, self-compassion is defined as extending compassion to oneself in instances of feeling inadequate and general suffering (Neff, 2016). Self-compassion was found to be negatively associated with symptoms of PGD and PTSD in bereaved individuals (Szőcs et al., 2022; Zhang et al., 2022).

However, little is known about how self-compassion is associated with symptoms of PGD and PTSD in bereaved individuals. Self-compassion can be loosely organized into three broad domains: (a) how people emotionally respond to suffering; (b) how they cognitively understand their predicament; and (c) how they pay attention to suffering (Neff, 2016, 2023). These components enable individuals to cultivate self-compassion, thereby reducing negative emotions and cognitions. Based on the definition of self-compassion, it may influence individuals’ stress responses through both cognitive and emotional pathways (Allen & Leary, 2010). Based on previous work, in the context of bereavement, perceived stigma, which is a common negative cognition among bereaved individuals, may be associated with how they interpret others’ evaluations of their grief, and can be understood as a form of cognitive appraisal of their social environment. In response to such appraisals, individuals may experience anger as an emotional reaction.

Perceived stigma describes the awareness of others’ stigmatizing attitudes and is a form of felt or subjective stigma (King et al., 2007). Perceived stigma following sudden death was a risk factor for suicidal thoughts and attempts (Pitman et al., 2017). Higher perceived stigma was associated with increased PGD symptoms in older Chinese shidu parents (Zhao et al., 2022) and with more severe PTSD symptoms in women receiving publicly funded substance abuse treatment (Matsumoto et al., 2021).

Anger, as one of the consequences of stigma or perceived stigma, is considered to be a better indicator of stigma (Frijda, 1986; Hansen & Sassenberg, 2011), and perceived stigma was found to be a significant predictor of anger (Else-Quest et al., 2009). Anger can be directed at the self or other people (Weiner, 1985). Individuals tend to experience self-directed anger when they attribute responsibility to themselves, and other-directed anger when they perceive others as responsible (Ellsworth & Tong, 2006; Hansen & Sassenberg, 2006). Self-directed anger and other-directed anger have different influences on both PGD and PTSD symptoms. Self-directed anger, closely associated with guilt, is positively linked to PGD and PTSD symptoms in bereaved people by suicide (Wagner et al., 2021) and road traffic accidents (Lenferink et al., 2024). Unlike the consistent association between self-directed anger and PGD and PTSD symptoms, findings on the impact of other-directed anger remain inconsistent. Researchers found that posttraumatic self-directed anger was associated with PTSD symptoms, whereas the association of posttraumatic other-directed anger with PTSD symptoms was found to be low (Orth & Maercker, 2009). However, a study involving victims of violent crime found that other-directed anger, but not self-directed anger, had an independent contribution to PTSD symptoms when all variables (i.e., shame and abuse in childhood) were considered together (Andrews et al., 2000). Thus, it is important to consider both self-directed and other-directed anger simultaneously so as to examine whether they have differential contributions to PGD and PTSD symptoms among bereaved individuals.

**Figure 1:**
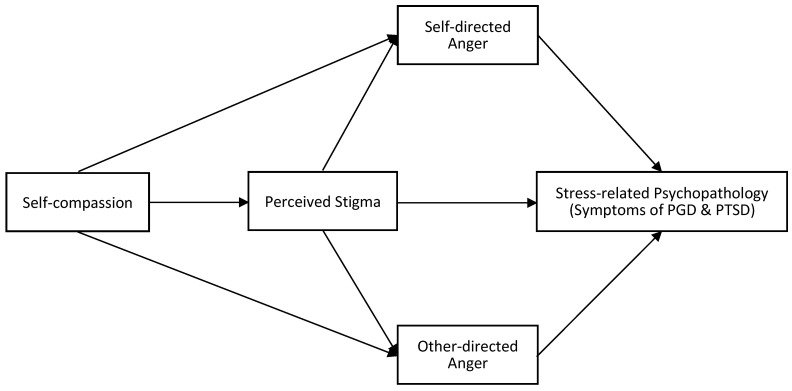
Hypothesized mediation model. Note: PGD, Prolonged Grief Disorder; PTSD, Posttraumatic Stress Disorder.

Given the potential role of perceived stigma and anger in the relationship between self-compassion and stress-related psychopathological symptoms in bereaved individuals, we proposed the following hypothesized model (as shown in Figure 1). Self-compassion may be associated with fewer negative cognitive responses to perceived stigma, which in turn decreases anger, a result of perceived stigma, and thereby alleviates the severity of psychopathological symptoms, represented by PGD and PTSD symptoms in the context of bereavement. Specifically, we hypothesize that: (a) self-compassion would be negatively associated with PGD and PTSD symptoms; (b) the association between self-compassion and PGD and PTSD symptoms would be indirectly associated via perceived stigma and anger (self-directed and other-directed), in the conceptual ordering specified by the model; (c) self-directed and other-directed anger would play distinct roles in the models of PGD and PTSD symptoms respectively. Clarifying these pathways enables researchers to gain a deep understanding of the development of PGD and PTSD in the bereaved population, and such understanding may provide guidance for strength-based and more targeted bereavement intervention.

## 2. Methods

### 2.1. Procedure and Participants

An online survey was conducted between July and November 2023. Participants were recruited through social networking websites and mobile applications. Eligible participants were aged 18 or older and had experienced the loss of a close person. Prior to beginning the survey, participants viewed a consent page outlining the study’s purpose, content, voluntariness, confidentiality, and data retention. Participants provided informed consent before accessing the questionnaire. Upon completion, participants received an electronic self-help manual as compensation. This study was approved by the Human Research Ethics Committee for Non-Clinical Faculties at the School of Psychology, Shenzhen University, prior to data collection (Approval No: [SZUPSY2023019]).

A total of 308 participants completed the survey. Nineteen participants were excluded from detailed data analysis because they were under 18 years of age (*n* = 8), completed the survey in under 5 min (*n* = 6) or showed patterned responses (*n* = 5). The final sample included 289 participants.

### 2.2. Measures

#### 2.2.1. Demographic and Loss-Related Variables

Participants provided demographic and loss-related information through self-report measures, including age and sex of the bereaved, relationship to the deceased, age and sex of the deceased, time since loss, and cause of death. Cause of death was assessed with a single self-report item asking participants to indicate the cause of the deceased person’s death. Response options included “chronic illness,” “acute illness,” “accident,” “suicide,” “COVID-19–related illness,” and “natural causes.”

#### 2.2.2. Self-Compassion

Self-compassion was assessed using the Self-Compassion Scale—Short Form (SCS-SF; Raes et al., 2011), validated in the Chinese version by Meng et al. (2019). The SCS-SF consists of 12 items that describe responses in difficult times, such as “When something painful happens, I try to take a balanced view of the situation”. Participants responded to a 5-point Likert scale ranging from 1 (*almost never*) to 5 (*almost always*). Negative-worded items were reverse-scored and the total score was calculated by summing all item scores. Higher scores indicate greater self-compassion. Cronbach’s α was 0.70 in this study.

#### 2.2.3. Prolonged Grief Symptoms

Prolonged grief symptoms were assessed using the 14-item International ICD-11 Prolonged Grief Disorder Scale (IPGDS; Killikelly et al., 2020), validated in Chinese bereaved people. It contains 13 symptom items (yearning, preoccupation, emotional distress and functioning impairment after the death of a close person) and one cultural screening item (e.g., “My grief would be considered worse than for others from my community or culture”). Participants indicated how often they experienced these symptoms in the past month on a 5-point Likert scale ranging from 1 (*almost never*) to 5 (*always*). A total score is summed across 13 symptom items. Higher scores indicated greater levels of symptoms. Cronbach’s α was 0.94 in the current sample.

#### 2.2.4. Posttraumatic Stress Symptoms

PTSD symptoms were measured using the Posttraumatic Stress Disorder Checklist for DSM-5 (PCL-5; Blevins et al., 2015), validated in Chinese bereaved individuals (Chen & Tang, 2021). It consists of 20 items, such as “repeated, disturbing dreams of the stressful experience”. The participants rated how bothered they had been by each item in the past month on a 5-point Likert scale ranging from 0 (*not at all*) to 4 (*extremely*). “The stressful experience’ referred to the death of a loved one.” Total scores were obtained by summing all items, with higher scores indicating greater PTSD symptom severity. The Cronbach’s α was 0.97 in the current sample.

#### 2.2.5. Perceived Stigma

The perceived stigma of the bereavement was measured using the 10-item stigma subscale of the Grief Experience Questionnaire (GEQ; Bailley et al., 2000). The Chinese version was developed through forward–backward translation by bilingual researchers and revised by a psychology professor. The stigma sub-scale includes items describing perceptions of others’ avoidance and lack of concern, capturing perceived rather than personal stigma (e.g., “Feel avoided by friends”). Responses were rated on a 5-point Likert scale ranging from 1 (*never*) to 5 (*almost always*), generating stigma subscale scores of 10 to 50. Higher scores reflect greater perceived stigma. In this study, the Cronbach’s α was 0.92 for the total scale. A one-factor CFA (WLSMV; ordered categorical items) supported a unidimensional structure of the Chinese version, with acceptable-to-good fit on incremental indices (CFI = 0.961, TLI = 0.950, SRMR = 0.049), although RMSEA was elevated (RMSEA = 0.143, 90% CI [0.126, 0.160]); standardized loadings ranged from 0.724 to 0.884.

#### 2.2.6. The Self and Other-Directed Anger

Self- and other-directed anger were assessed using an 8-item Post-Bereavement Anger Questionnaire (PAQ), adapted from the Posttraumatic Anger Questionnaire (Orth & Maercker, 2009). The original PAQ has previously been applied in bereaved samples to assess anger (Lenferink et al., 2024). The original 20-item measure covers five anger domains. We only focus on the post-bereavement anger directed at (a) the self (Self-directed anger, e.g., “I was angry at myself because I still feel weak and vulnerable because of the bereavement event”) and (b) other people (Other-directed anger, e.g., “I was angry at other people because they did not prevent the bereavement event”). Translation followed a blind forward–backward procedure by bilingual researchers. Items are answered on a 6-point Likert scale ranging from 0 (*never*) to 5 (*very often*). The total score was calculated by summing all item scores. Higher subscale scores indicate greater levels of specific anger. Cronbach’s α for the subscales in this research is 0.90 for self-directed anger and 0.88 for other-directed anger.

### 2.3. Statistical Analysis

Descriptive statistics (mean and standard deviation) were reported for all variables. Given that our conceptual model specified perceived stigma followed by two parallel mediators (namely self-directed anger and other-directed anger), we used PROCESS Model 81 to estimate the corresponding serial indirect associations in a single model. We estimated PGD and PTSD in separate models using an identical mediator structure and covariate set to facilitate comparison across outcomes (Hayes, 2013). The mediation analyses were implemented with 5000 bootstrap samples and 95% bias-corrected confidence intervals (CIs). Effects were considered significant if the 95% CIs did not include zero. Multicollinearity was evaluated using tolerance and variance inflation factors (VIFs). No problematic multicollinearity was detected (tolerance = 0.585–0.924; VIF = 1.082–1.710), indicating that the mediators and covariates could be included simultaneously in the models. All analyses were performed using SPSS 27.0 (IBM Corporation, Armonk, NY, USA).

In both the PGD and PTSD symptoms models, we controlled for demographic and loss-related variables based on previous research (Zhou et al., 2018; Doering et al., 2022), and coded them as follows: demographic and loss-related variables included age of the bereaved, sex of the bereaved (1 = female, 0 = male), relationship between the bereaved and the deceased (1 = first-degree relative [parent, child, spouse], 0 = others [grandparent, sibling, other relative, friend, other]), age of the deceased, sex of the decreased (1 = female, 0 = male), time since loss in years, cause of death (1 = sudden death [acute illnesses, accidents, suicides, COVID-19-related illness, respiratory failure], 0 = non-sudden death [chronic illness, natural causes]).

## 3. Results

### 3.1. Sample Characteristics

Table 1 presents the demographic and loss-related characteristics of the 289 participants (70.2% women; Mage = 42.27 ± 11.37 years). Participants were bereaved 7.85 ± 9.25 years ago, and over half (50.2%) of the participants had lost a parent, a child, or a spouse. The majority (80.6%) of the deaths were natural deaths.

### 3.2. Correlations Among Key Variables

As shown in Table 2, self-compassion was significantly negatively correlated with PGD and PTSD symptoms, perceived stigma, self-directed anger, and other-directed anger. Perceived stigma, self-directed anger, and other-directed anger were significantly positively associated with PGD and PTSD symptoms.

### 3.3. Serial Mediation by Perceived Stigma and Anger

**Figure 2:**
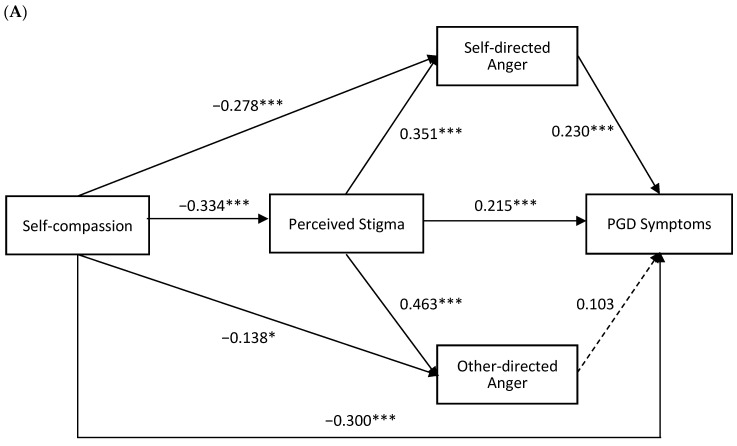

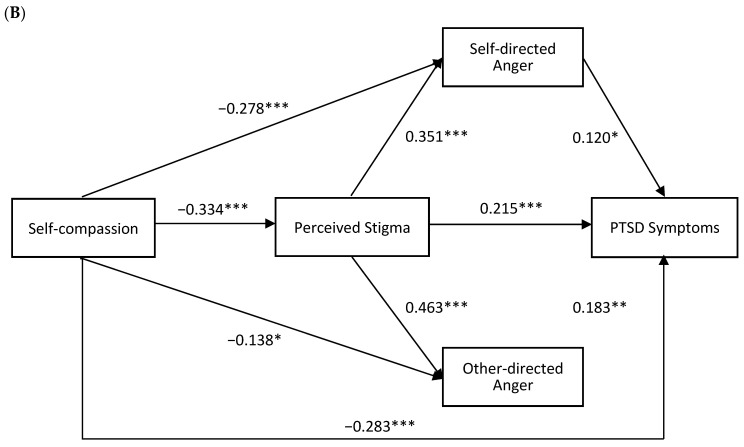
The PGD symptoms model (panel (**A**)) and the PTSD symptoms model (panel (**B**)) (*N* = 289). Note: PGD, Prolonged Grief Disorder; PTSD, Posttraumatic Stress Disorder. Covariates are age and gender of the bereaved, relationship between the bereaved and the deceased, age of the deceased, time since loss, and cause of death. The values shown are standardized path coefficients. Black solid lines refer to significant paths (*p* < 0.05) and dashed lines refer to nonsignificant paths (*p* > 0.05). * *p* < 0.05, ** *p* < 0.01, *** *p* < 0.001.

To examine the mediating roles of perceived stigma, self-directed anger and other-directed anger between self-compassion and PGD and PTSD symptoms, two path analyses were conducted, controlling for demographic and loss-related covariates (see Figure 2). Table 3 shows the direct, indirect and total effects of the serial mediation model.

In the PGD symptoms model (R^2^ = 0.4371), self-compassion was significantly negatively associated with PGD symptoms. After including perceived stigma and both anger types, the direct effect of self-compassion on PGD symptoms remained significant (Effect = −0.6409, 95% CI = [−0.8591, −0.4227]). For indirect pathways, the indirect effect of self-compassion via perceived stigma was significant (Effect = −0.1528, 95% CI = [−0.2458, −0.0695]). The indirect effect of self-compassion via self-directed anger (Effect = −0.1360, 95% CI = [−0.2518, −0.0439]) and self-compassion via perceived stigma and self-directed anger (Effect = −0.0573, 95% CI = [−0.0992, −0.0214]) were significant. However, the indirect effect of self-compassion via perceived stigma and other-directed anger (Effect = −0.0338, 95% CI = [−0.0831, 0.0113]) and self-compassion via other-directed anger (Effect = −0.0302, 95% CI = [−0.0829, 0.0101]) were not significant.

In the PTSD symptoms model (R^2^ = 0.4637), self-compassion was significantly negatively associated with PTSD symptoms. After including mediators, the direct effect remained significant (Effect = −0.8719, 95% CI = [−1.1798, −0.5641]). For indirect pathways, the indirect effect of self-compassion via perceived stigma was significant (Effect = −0.3126, 95% CI = [−0.4789, −0.1732]). The indirect effect of self-compassion via perceived stigma and other-directed anger (Effect = −0.0872, 95% CI = [−0.1565, −0.0280]) and self-compassion via other-directed anger (Effect = −0.0779, 95% CI = [−0.1692, −0.0106]) were significant. However, the indirect effect of self-compassion via self-directed anger (Effect = −0.1028, 95% CI = [−0.2338, 0.0053]) and self-compassion via perceived stigma and self-directed anger (Effect = −0.0433, 95% CI = [−0.0899, 0.0027]) were not significant.

## 4. Discussion

This study is the first to explore distinct mediation pathways involving perceived stigma and two types of anger (self-directed and other-directed anger) in the relationship between self-compassion and PGD and PTSD symptoms. The results confirmed that self-compassion was negatively associated with PGD and PTSD symptoms. More importantly, the study observed two differentiated indirect pathways: perceived stigma and self-directed anger formed an indirect pathway linking self-compassion and PGD symptoms, while perceived stigma and other-directed anger formed an indirect pathway linking self-compassion and PTSD symptoms. These distinct pathways deepen understanding of how self-compassion relates to bereavement-related outcomes and align with stress and coping theories, which emphasize the role of cognitive appraisal and emotional responses in shaping adjustment to stress (Lazarus & Folkman, 1984). Given the cross-sectional design, the mediation models are interpreted as statistical associations and do not imply temporal or causal direction.

### 4.1. Self-Compassion and PGD and PTSD Symptoms

The results showed that self-compassion was significantly negatively associated with PGD and PTSD symptoms, consistent with our hypotheses and aligning with prior work (Inwood & Ferrari, 2018; Szőcs et al., 2022; Zhang et al., 2022). Notably, the direct associations between self-compassion and both outcomes remained significant after accounting for perceived stigma and anger, suggesting that additional pathways beyond the tested indirect effects may also be involved. PGD and PTSD often show substantial overlap in bereaved populations (Komischke-Konnerup et al., 2021; Eddinger et al., 2021), and they were also strongly correlated in the present sample; accordingly, the differentiated indirect patterns observed here should be interpreted cautiously and examined in more diverse and representative samples. As an adaptive emotion regulation strategy, self-compassion enables individuals to treat their own suffering with kindness and acceptance, thereby reducing experiential avoidance and self-critical tendencies, ultimately mitigating grief- and trauma-related distress (Neff, 2016; Inwood & Ferrari, 2018).

These findings suggest that self-compassion-based interventions, such as self-compassion writing or mindfulness training (Szőcs et al., 2022), may be beneficial for PGD and PTSD symptoms. The present study provides preliminary evidence for developing self-compassion-oriented grief interventions.

### 4.2. Mediating Role of Perceived Stigma

Our results also revealed that perceived stigma was involved in an indirect association linking self-compassion and both PGD and PTSD symptoms, consistent with prior studies (Matsumoto et al., 2021; Pitman et al., 2017; Zhao et al., 2022). Self-compassion may be related to lower levels of perceived stigma. For example, the emotional component of self-compassion (greater self-kindness and less self-judgment) is often conceptualized as an emotion-regulation strategy and may be associated with less avoidance and entanglement with negative emotions and better regulation skills (Yela et al., 2022; Miyagawa et al., 2022; Inwood & Ferrari, 2018). When bereaved individuals internalize perceived stigma, they may feel shame or guilt about their bereavement experience or grieving process, which may be associated with greater severity of grief symptoms. Additionally, from the cognitive perspective, perceived stigma is associated with amygdala reactivity linked to threat and fear (Hatzenbuehler et al., 2022). By re-experiencing such fear and threat, individuals might recall unwanted memories of the bereavement event, potentially relating to severe intrusive symptoms of PTSD. Moreover, perceived stigma may deter help-seeking behaviors (Mittal et al., 2013). Overall, perceived stigma was consistently involved in all indirect paths, underscoring its transdiagnostic role across PGD and PTSD; it may represent a promising target for future intervention research.

### 4.3. Distinct Roles of Self-Directed and Other-Directed Anger

#### 4.3.1. Pathway via Perceived Stigma and Self-Directed Anger

Self-directed anger showed an indirect association between self-compassion and PGD symptoms. As an adaptive emotion regulation strategy, self-compassion helps manage negative emotions (e.g., self-directed anger; Allen & Leary, 2010; Diedrich et al., 2014), and it helps bereaved individuals acknowledge painful feelings with kindness and understanding (Neff, 2003). Individuals with higher self-compassion may acknowledge self-directed anger through mindful awareness rather than being overwhelmed. Extending kindness helps prevent harsh self-criticism that often accompanies self-directed anger (Gilbert & Procter, 2006). Moreover, self-directed anger is strongly associated with guilt, which predicts greater PGD symptoms (Wagner et al., 2021; Boelen & Lenferink, 2022).

Perceived stigma and self-directed anger formed a significant indirect pathway linking self-compassion and PGD symptoms, while the indirect pathway regarding other-directed anger was not significant. Individuals perceiving higher stigma while blaming themselves may also report higher levels of self-directed anger. According to the self-stigma framework, perceived stigma may be associated with self-stigma, where individuals internalize societal prejudices and develop negative self-concepts (Corrigan, 1998). This process may be linked to self-directed anger and be associated with self-blame and guilt, which are associated with PGD symptoms. Fortunately, the nonjudgmental and compassionate attitude inherent in self-compassion may allow bereaved individuals to process stigma without triggering self-criticism, thereby reducing self-directed anger (Wong et al., 2019) and easing PGD symptoms.

The indirect pathway from self-compassion to PTSD symptoms via self-directed anger was not significant. Some evidence may help explain why these two indirect paths differ. Self-directed anger, rather than other-directed anger, was associated with PGD symptoms. Prior work suggests that self-directed anger elicits greater motivation to suppress one’s emotions (Ellsworth & Tong, 2006), which may contribute to prolonged separation and traumatic distress (Boelen & Lenferink, 2022).

#### 4.3.2. Pathway via Perceived Stigma and Other-Directed Anger

The indirect effect of self-compassion on PTSD symptoms via other-directed anger was significant. This may reflect the role of self-compassion, particularly its common humanity component, in being associated with lower levels of other-directed anger. Self-compassion may foster a broader understanding of suffering, promoting awareness of shared human imperfection and empathy for others, which may help mitigate other-directed anger (Neff & Pommier, 2013). Empirical studies also demonstrate an inverse association between self-compassion and anger in mindfulness-based interventions (Robins et al., 2012; Smeets et al., 2014).

Perceived stigma and other-directed anger were involved in a significant indirect association linking self-compassion and PTSD symptoms, whereas the pathway involving self-directed anger was not significant. According to stress and coping theories, perceived stigma may function as a stressor, and individuals may appraise their situation as threatening, which can result in outward anger (i.e., other-directed anger) and exacerbate PTSD symptoms (Lazarus & Folkman, 1984). Emotion-Focused coping, such as focusing on anger, is often viewed as a maladaptive coping strategy (Lazarus & Folkman, 1984; Cooper & Quick, 2017). These reactions (e.g., escape and avoidance; Dewe & Cooper, 2007) have been associated with adverse outcomes (e.g., PTSD symptoms; Folkman & Moskowitz, 2004). Self-compassion may help bereaved individuals embrace common humanity and avoid over-identification with uncomfortable thoughts and emotions, which may be associated with lower other-directed anger (Wu et al., 2019).

However, the indirect effect of self-compassion on PGD symptoms via other-directed anger was not significant. The finding that other-directed anger was independently associated with PTSD symptoms may be explained by the following factors. Bereavement represents a traumatic event, and the relation between other-directed anger and PTSD symptoms may stem from trauma-related anger (e.g., anger against individuals who did not prevent the occurrence of the traumatic event) or increased non-trauma-related anger (e.g., anger at the workplace or in the family; Orth & Wieland, 2006). Moreover, anger toward others independently predicts PTSD symptoms (Andrews et al., 2000). Although another study found that the association of self-directed posttraumatic anger with PTSD symptoms was stronger than that of other-directed posttraumatic anger with PTSD symptoms (Orth & Maercker, 2009), this discrepancy could result from the different meanings of other-directed anger. In the study by Orth and Maercker, the word ‘other-directed posttraumatic’ refers to anger toward other people except for anger directed at the self, the justice system, people held accountable for the potential traumatic event and a desire for revenge against those held responsible. However, when considering anger toward all other people except for the self, the association of other-directed anger would be stronger.

Prior work suggests that self-directed anger may reflect heightened perceived control, wherein individuals believe they should have been able to prevent the adverse event, leading to guilt and self-blame, which in turn exacerbate PGD symptoms (Wagner et al., 2021). In contrast, other-directed anger may reflect attribution to uncontrollable external factors, which may be linked to heightened perceived threat and higher levels of PTSD symptoms (Boelen et al., 2022). Future research should further examine the underlying mechanisms through which perceived control operates in bereaved individuals.

### 4.4. Limitations

Several limitations warrant attention. First, the cross-sectional data cannot establish temporal precedence among variables (Stone-Romero & Rosopa, 2011). Future work should employ a longitudinal methodology and examine the directionality of the observed effects. Thus, the proposed mediation pathways should be interpreted as statistical associations consistent with the hypothesized model, and reverse or alternative directional models cannot be ruled out. Future research should also examine potential moderators, particularly time since loss, to determine whether the observed associations differ across stages of bereavement. Second, all variables were measured retrospectively, which may be biased by individuals’ subjective emotional status. Future studies should consider methods with higher ecological validity (e.g., ecological momentary assessment) to reduce retrospective recall bias and to capture how self-compassion, perceived stigma, and anger covary with PGD and PTSD symptoms in daily life (Boelen & Lenferink, 2022; Li et al., 2020). Third, although most measures showed good internal consistency in the present sample, the internal consistency of the SCS-SF was modest (α = 0.70). This modest reliability may attenuate associations involving self-compassion and should be considered when interpreting the magnitude of effects. In addition, the scales of perceived stigma and post-bereavement anger used in this study would benefit from more systematic psychometric validation and refinement in independent bereaved samples to further strengthen evidence for their reliability and validity in bereaved populations. Finally, the sample was predominantly female and recruited online, which may limit generalizability to male bereaved individuals and to groups with lower internet access or digital literacy. In addition, participants were Chinese bereaved adults; therefore, cultural and gender norms surrounding bereavement, emotional expression, and stigma may constrain the applicability of the findings to other cultural contexts. Future studies should consider more diverse and community-based sampling strategies to improve representativeness.

## 5. Conclusions

Notwithstanding these considerations, this study provides empirical support for a model in which self-compassion is associated with lower PGD and PTSD symptoms by reducing perceived stigma and anger. This study is the first to show evidence of distinct indirect pathways linking self-compassion with PGD and PTSD symptoms through perceived stigma and different forms of anger. These findings suggest that clinical interventions for bereaved individuals may benefit from integrating self-compassion-based strategies with conventional cognitive behavioral therapy. Given the differential roles of anger, intervention strategies should be appropriately tailored. For PGD, targeting self-directed anger and guilt is essential, while for PTSD, addressing other-directed anger may be more relevant. Self-compassion may address both emotional pathways by fostering self-kindness and enhancing a sense of common humanity.

## Figures and Tables

**Table 1:** Demographic and loss-related characteristics (*N* = 289).

Variable	*N* (M)	% (SD)
Sex		
Female	203	70.2
Male	86	29.8
Age	42.27	11.37
Sex of deceased		
Female	119	41.2
Male	170	58.8
Age of deceased	67.62	17.74
Time since loss (in years)	7.85	9.25
Relationship with the deceased		
Parent	134	46.4
Grandparent	77	26.6
Spouse	10	3.5
Child	1	0.3
Sibling	17	5.9
Other relative ^a^	26	9.0
Friend	19	6.6
Others ^b^	5	1.7
Cause of death ^c^		
Sudden death	157	54.3
Non-sudden death	132	45.7

Note: *N*, Number; %, Percentage; M, Mean; SD, Standard Deviation. ^a^ Other relative includes uncle/aunt (*n* = 18), cousin (*n* = 2), and other extended family members (*n* = 6). ^b^ Other includes teacher (*n* = 1), neighbor (n = 1), and other non-family members (*n* = 3). ^c^ Cause of death includes acute illnesses (*n* = 77), accidents (*n* = 26), suicides (*n* = 8), and COVID-19-related pneumonia and respiratory failure (*n* = 5); Non-sudden death includes long-term illnesses (*n* = 128) and natural causes (*n* = 29).

**Table 2:** Descriptive statistics and bivariate correlations of key variables (*N* = 289).

Variables	1	2	3	4	5	6
1. Self-compassion	–					
2. Perceived stigma	−0.379 ***	–				
3. Self-directed anger	−0.404 ***	0.477 ***	–			
4. Other-directed anger	−0.315 ***	0.533 ***	0.524 ***	–		
5. PGD symptoms	−0.476 ***	0.486 ***	0.513 ***	0.424 ***	–	
6. PTSD symptoms	−0.499 ***	0.559 ***	0.454 ***	0.492 ***	0.756 ***	–
*M (SD)*	38.15 (5.48)	8.46 (3.58)	6.94 (4.87)	4.42 (4.01)	20.10 (11.69)	18.92 (16.90)
Actual range	12–54	5–25	0–20	0–17	0–56	0–69
Cronbach’s α	0.70	0.92	0.90	0.88	0.94	0.97

Note: PGD, Prolonged Grief Disorder; PTSD, Posttraumatic Stress Disorder. *** *p* < 0.001.

**Table 3:** Direct and indirect effects from self-compassion, perceived stigma, self-directed anger and other-directed anger to PGD and PTSD symptoms.

Pathway	Effects	SE	LLCI	ULCI
**1. Self-compassion → PGD symptoms**				
Direct	−0.6409	0.1108	−0.8591	−0.4227
Indirect	−0.4100	0.0672	−0.5478	−0.2826
Self-compassion → Perceived stigma → PGD symptoms	−0.1528	0.0451	−0.2458	−0.0695
Self-compassion → Perceived stigma → Self-directed anger → PGD symptoms	−0.0573	0.0197	−0.0992	−0.0214
Self-compassion → Perceived stigma → Other-directed anger → PGD symptoms	−0.0338	0.0239	−0.0831	0.0113
Self-compassion → Self-directed anger → PGD symptoms	−0.1360	0.0539	−0.2518	−0.0439
Self-compassion → Other-directed anger → PGD symptoms	−0.0302	0.0236	−0.0829	0.0101
**2. Self-compassion → PTSD symptoms**				
Direct	−0.8719	0.1564	−1.1798	−0.5641
Indirect	−0.6238	0.0929	−0.8143	−0.4484
Self-compassion → Perceived stigma → PTSD symptoms	−0.3126	0.0775	−0.4789	−0.1732
Self-compassion → Perceived stigma → Self-directed anger → PTSD symptoms	−0.0433	0.0233	−0.0899	0.0027
Self-compassion → Perceived stigma → Other-directed anger → PTSD symptoms	−0.0872	0.0326	−0.1565	−0.0280
Self-compassion → Self-directed anger → PTSD symptoms	−0.1028	0.0609	−0.2338	0.0053
Self-compassion → Other-directed anger → PTSD symptoms	−0.0779	0.0410	−0.1692	−0.0106

Note: Effects are unstandardized estimates. SE, bootstrap standard error; LLCI, lower bound of the 95% bootstrap confidence interval; ULCI, upper bound of the 95% bootstrap confidence interval. Self-directed anger, Post-bereavement Anger Questionnaire—anger towards the self; Other-directed anger, Post-bereavement Anger Questionnaire—anger towards other people; PGD, Prolonged Grief Disorder; PTSD, Posttraumatic Stress Disorder. Indirect effects were estimated using bootstrap resampling (5000 samples) and were considered statistically significant when the 95% bootstrap confidence interval (Boot LLCI–Boot ULCI) did not include zero. Covariates were age and gender of the bereaved, relationship between the bereaved and the deceased, age and gender of the deceased, time since loss and cause of death.

## Data Availability

For ethical and privacy reasons, the de-identified dataset is not publicly available. It may be obtained from the corresponding author upon reasonable request, with ethics approval and a data use agreement.
